# TPR1, a novel rifampicin derivative, demonstrates efficacy alone and in combination with doxycycline against the NIAID Category A priority pathogen *Francisella tularensis*

**DOI:** 10.1093/jacamr/dlab058

**Published:** 2021-05-04

**Authors:** Jason E Cummings, Keaton W Slayden, Richard A Slayden

**Affiliations:** Department of Microbiology, Immunology and Pathology, Colorado State University, Fort Collins, CO, USA; Department of Microbiology, Immunology and Pathology, Colorado State University, Fort Collins, CO, USA; Department of Microbiology, Immunology and Pathology, Colorado State University, Fort Collins, CO, USA

## Abstract

**Background:**

*Francisella tularensis* is a highly virulent and contagious Gram-negative intracellular bacterium that causes the disease tularaemia in mammals and is classified as a Category A priority pathogen.

**Methods:**

We utilized a systematic analysis of antibacterial potency, extent of dissemination by analysis of bacterial burden in a secondary vital organ, and survival rates to assess the efficacy of a novel rifampicin derivative, TPR1. The efficacy of TPR1 was evaluated alone and in combination with the standard of care drug, doxycycline, against type A *F. tularensis* Schu S4 using a lethal pulmonary model of infection in mice.

**Results:**

TPR1 has an MIC value range of 0.125–4 mg/L against reference laboratory strain Schu S4 and a panel of clinical strains. TPR1 alone reduced the bacterial burden in the lungs and spleen at 40 mg/kg and 80 mg/kg, and no antagonism was observed when co-administered with doxycycline. Dosing at 40 mg/kg doxycycline reduced the bacterial burden by 1 log_10_ cfu in the lungs and 4 log_10_ cfu in the spleen in comparison to untreated controls. Co-administration of TPR1 and doxycycline demonstrated efficacy upon treatment withdrawal after 4 days of treatment, and 100% survival.

**Conclusions:**

Significantly, TPR1 demonstrated efficacy when delivered alone and in combination with doxycycline, which provides compelling evidence of a superior treatment strategy that would normally rely on a single chemotherapeutic for efficacy. In addition, this work substantiates the use of rifampicin derivatives as a platform for the development of novel treatments to other bacterial agents in addition to tularaemia.

## Introduction

*Francisella tularensis* is a highly virulent and infectious Gram-negative intracellular bacterium that causes the disease tularaemia in mammals and is often fatal when acquired by inhalation.^1^^,^^2^ In addition to being an emerging difficult-to-treat pathogen, NIAID has classified *F. tularensis* as a Category A priority pathogen. Tularaemia is distributed worldwide including North America, Europe, Australia, Japan, China, the Middle East and Russia.^3^ Tularaemia outbreaks are commonly reported in these endemic regions and typically associated with contaminated water or soil, wild and domestic animals, and common arthropod vectors.^3^ Thus, there is an important and continuing need for new treatments for tularaemia.

Streptomycin and gentamicin are considered the first-line clinical drugs, and secondary treatment regimens for *F. tularensis* infections utilize doxycycline and ciprofloxacin.^4^ The use of traditional chemotherapeutics including streptomycin and gentamicin is often limited because of toxic side effects, and neither can be orally administered. Fluoroquinolones are the first-line oral treatment for type B tularaemia, however, their role in treating severe type A infections has been less evaluated. Despite the availability of drugs such as the aminoglycosides, chloramphenicol, fluoroquinolones and tetracyclines, relapse of disease is found in 10% of the cases and can result in a mortality as high as 40%.^4–6^ In animal studies, gatifloxacin, moxifloxacin and ciprofloxacin prevented disease during the treatment period, but significant failure rates occurred after the withdrawal of therapy.^3^ Since existing drugs have limited potency and efficacy, and it is unknown whether drug-resistant organisms might be used in an intentional release, it is prudent to develop novel chemotherapeutics with proven modes of action and reduced side effects that can be co-administered with standard of care (SoC) drugs to treat acute infections and prevent relapse of disease.

Doxycycline and rifampicin have been shown to have activity against *F. tularensis.*^7^ Although the activity of these drugs against *F. tularensis* has been reported, they had limited efficacy, and drug resistance has been observed *in vitro*.^5^^,^^8^  *Ex vivo* studies have demonstrated that rifampicin penetrates well within eukaryotic cells and demonstrates activity against intracellular *F. tularensis,* but development of resistance and frequent disease relapse is observed upon withdrawal of drug treatment.^5^^,^^7^ Similarly, doxycycline has been shown to be efficacious against *F. tularensis* but has been associated with frequent clinical disease relapses.^1^ There are numerous drug treatment guidelines for *F. tularensis,* with the recommendation to administer streptomycin and gentamicin for at least 10–14 days as well as tetracyclines and chloramphenicol for at least 14–21 days.^3^^,^^7^^,^^9^ In severe cases, two drug combinations are recommended to prevent relapse and drug resistance.

A historically successful approach in drug discovery is repurposing or to utilize a known pharmacophore as the foundation for derivatization to create a new drug.^10–12^ The identification and use of a derivative of a widely used broad-spectrum agent promises to improve management of emerging pathogens, such as *F. tularensis.* The bacterial DNA-dependent RNA polymerase is a widely exploited drug target in bacteria, making rifampicin an ideal pharmacophore as a foundation for new derivatives. In this study, we assessed the use of a novel rifampicin derivative, TPR1, alone and in combination with doxycycline to reduce bacterial burden, prevent bacterial dissemination and relapse, and increase survival. *In vitro* results show TPR1 being equally active against a diverse panel of *F. tularensis* strains that included clinical isolates and reference laboratory strains. The MIC value of TPR1 was 0.125–4 mg/L, which is comparable to current SoC drugs used to treat infections caused by *F. tularensis.*^7^ TPR1 also demonstrated efficacy in a mouse model of *F. tularensis* infection when administered alone at 80 mg/kg and in combination with doxycycline at sub-efficacious doses of 40 mg/kg as determined by a reduction in bacterial burden in the lungs and spleen with no observable disease relapse. This substantiates the use of the rifampicin derivative, TPR1, alone or in combination with doxycycline for the treatment of infections caused by *F. tularensis* that will provide a durable cure without relapse or the emergence of drug resistance associated with single-drug treatments.

## Methods

### MIC of TPR1

To assess activity of TPR1 (supplied by Palisades Therapeutics/Pop Test Oncology LLC) against *F. tularensis,* MIC assays were performed using the *F. tularensis* laboratory reference strain Schu S4 and a panel of clinical strains. MICs were determined via CLSI guidelines M07/M45 as previously described.^13^  *F. tularensis* was grown to mid-log stage and diluted to provide an inoculum of ∼10^5^ cells/mL when dispensed into a 96-well microtitre drug plate. Compounds in the drug plates were tested at 2-fold serial concentrations from 0.0156–32 mg/L in triplicate. OD readings were obtained after 18–20 h of incubation at 37 °C and MIC defined as the first concentration to exhibit no growth.

### Determination of cytotoxicity

HepG2 and HeLa cells were grown in Eagle’s Minimum Essential Medium (ATCC) supplemented with 10% (v/v) fetal bovine serum (cEMEM). THP-1 cells were grown in RPMI1640 with 10% fetal bovine serum and 0.05 mM 2-mercaptoethanol supplementation. Drug plates were prepared with 1:2-fold dilutions starting with 128 mg/L and ending with 0.0625 mg/L in respective medium. Drug medium was added to washed cells and incubated at 37 °C with 5% CO_2_ for 24 h. Medium was removed and replaced with fresh medium. Then, 0.01 mL of 12 mM methylthiazol tetrazolium (MTT, Sigma) was added to the plates and cells were incubated for an additional 4 h at 37 °C with 5% CO_2_. Detergent solution (0.1 mL of 0.1 g/mL SDS in 0.01 M HCl) was added to each well and plates were incubated for an additional 4 h at 37 °C with 5% CO_2_. Absorbance of plates were measured at 570 nm to determine percentage growth reduction over the concentration series. Values were plotted using Prism software and IC_50_ values (lethal concentration causing 50% loss of cell viability) determined by linear regression analysis.

### Efficacy and survival studies

Seven- to nine-week-old female BALB/c mice were purchased from Charles River Laboratories (Jackson Laboratories, Bar Harbor, ME, USA). All mice were housed in micro-isolator cages in the laboratory animal resources facility or in the Infectious Diseases Research Complex BSL-3 facility at Colorado State University and were provided sterile water and food ad libitum. All research involving animals was conducted in accordance with animal care and use guidelines and animal protocols were approved by the Animal Care and Use Committee at Colorado State University. Mice were anaesthetized with a mixture of ketamine/xylazine to achieve 100 mg/kg ketamine and 10 mg/kg xylazine in a volume of 0.1 mL per mouse delivered intraperitoneally. Mice were infected with 100 cfu Schu S4 delivered intranasally as previously described.^2^^,^^13^^,^^14^ Mice were monitored for morbidity and mortality twice daily.

TPR1 was dissolved in DMSO to a final concentration of 160 mg/mL, resulting in a dosing stock that was further diluted in sterile water dropwise while mixing to a final concentration of 8 mg/mL. Treatments were delivered at 100 µL per 20 g bodyweight by mouth (PO) for 40 mg/kg or with 200 µL per 20 g bodyweight PO for 80 mg/kg. Doxycycline (160 mg) was dissolved into 20 mL PBS and filter sterilized. Mice were treated with 100 µL per 20 g bodyweight PO.

The study design consisted of five experimental groups: Group 1, untreated control (5% DMSO/water PO twice a day); Group 2, doxycycline (PBS 40 mg/kg PO twice a day); Group 3, TPR1 (5% DMSO/water 40 mg/kg PO twice a day); Group 4, doxycycline and TPR1 co-treatment (TPR1, 5% DMSO/water 40 mg/kg PO twice a day; doxycycline, PBS 40 mg/kg PO twice a day); Group 5, TPR1 (5% DMSO/water 80 mg/kg PO twice a day). Experimental endpoints were 96 h post-infection, based on the mean time untreated animals become moribund, and 28 days post-infection, representing 7 × the moribund mean time to assess disease relapse. At each 96 h timepoint, representative animals from each group were sacrificed, and bacterial burden in the lungs and spleen were determined by plating and enumeration of cfu. The remaining animals in each group were monitored until moribund or the study endpoint if appropriate, and bacterial burden was assessed in vital organs.

### Ethics statement

Use of vertebrate animals at Colorado State University is conducted under AAALAC approval OLAW number A3572-01 under file with NIH. Animals are housed in ABL-3 facility under supervision by full-time veterinarians per American Veterinary Medical Association guidelines.

## Results and discussion

### TPR1 is a potent inhibitor of F. tularensis with low cytotoxicity

TPR1 was screened against a diverse panel of *F. tularensis* strains consisting of laboratory and clinical strains that are considered representative of the drug susceptibility spectrum associated with clinical infections. TPR1 has an MIC range of 0.125–4 mg/L against the laboratory reference strain and a panel of clinical strains with various susceptibilities to SoC drugs (Table 1). This MIC value and inhibition are characteristic of clinical drugs with bactericidal activity against *F. tularensis*, which is within the range for a drug to have efficacy. Most significantly, TPR1 demonstrated potency comparable to current clinically used drugs to treat *F. tularensis* infections with no observable spontaneous drug resistance. For example, the MICs determined for various clinical strains of *F. tularensis* with streptomycin and gentamicin fall into a range of 2–4 mg/L and 1–2 mg/L, respectively. To assess the potential safety window of TPR1, cytotoxicity was evaluated in HepG2, THP-1 and HeLa cells. The LC_50_ for TPR1 was determined to be >128 mg/L in each of these cell lines, which is comparable to other clinical drugs and represents a significant difference in the selective index (SI) between tissue toxicity and bactericidal dose (Table 1).

**Table 1.** MIC and cytotoxicity

	TPR1	Rifampicin	Doxycycline	Gentamicin
MIC, mg/L				
* F. tularensis* Schu S4 Submaster Cell Bank (BEI NR-10492)	4	0.5	1	1
* F. tularensis* Schu S4 FSC237 (BEI NR-643)	2	0.25	2	1
* F. tularensis* WY96-3418 (BEI NR-644)	0.125	0.0625	0.125	0.25
* F. tularensis* MA00-2987 (BEI NR-645)	4	0.5	2	1
* F. tularensis* KY99-3387 (Sub-species type B) (BEI NR-647)	4	0.5	2	1
* F. tularensis* OR96-0246 (Sub-species type B) (BEI NR-648)	2	0.25	0.5	0.5
Cytotoxicity (LD_50_), mg/L				
HepG2	>128	N/A	N/A	N/A
THP-1	>128	N/A	N/A	N/A
HeLa	>128	N/A	N/A	N/A

N/A, not applicable.

### TPR1 demonstrates efficacy and controls dissemination

**Figure 1.**
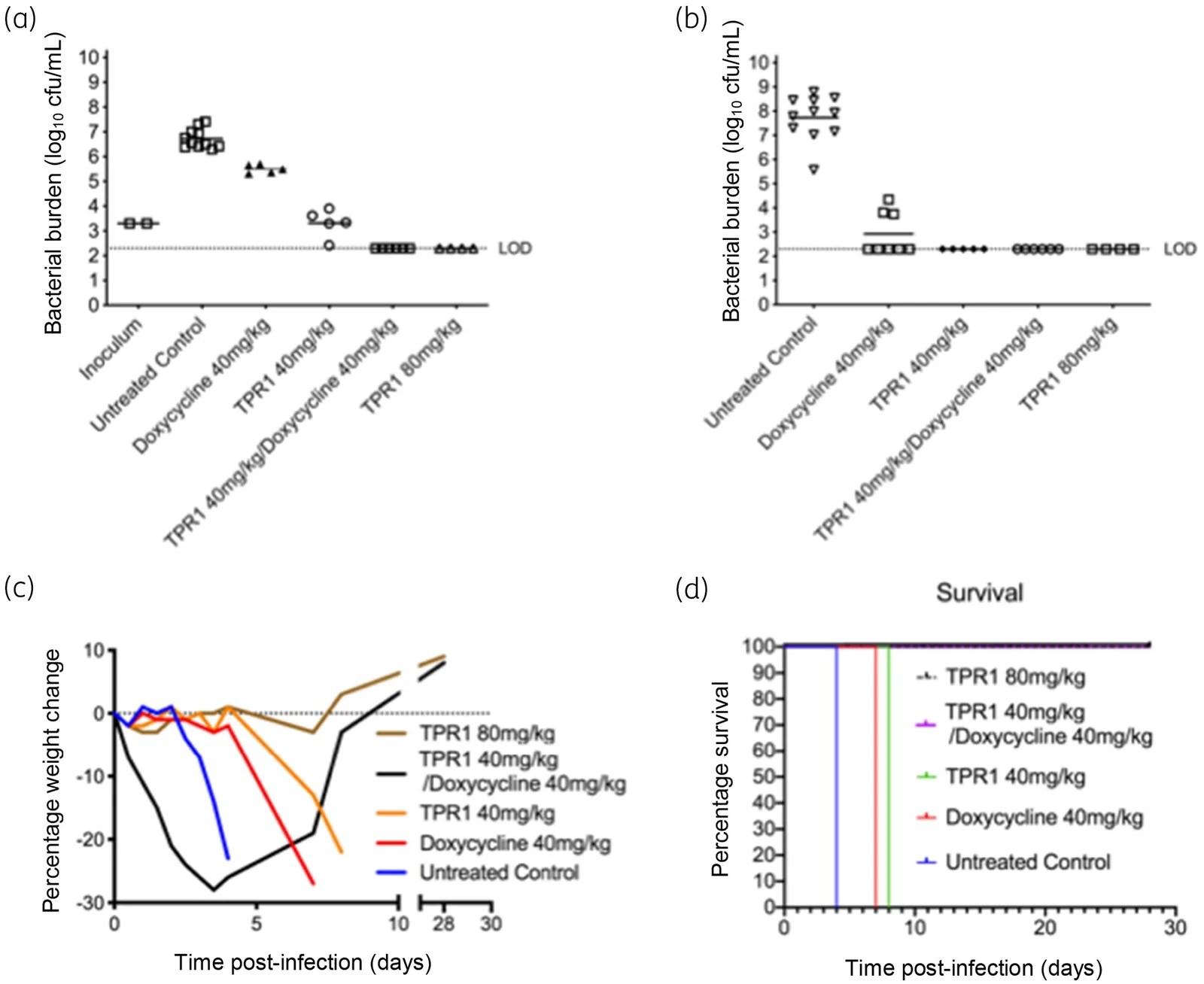
Efficacy in murine model of tularaemia. Bacterial burdens in the lung and spleen during treatment with TPR1. *F. tularensis* cfu (log_10_ cfu/mL) recovered from the (a) lungs and (b) spleen at Day 4 of treatment. (c) Percentage change in animal body weight and (d) survival of mice treated with TPR1 or doxycycline, and TPR1 doxycycline co-administration.

The efficacy of TPR1 was determined in the standard *F. tularensis* murine model of infection. For these studies, mice were infected with *F. tularensis* Schu S4 and administered TPR1 once a day for 5 days beginning Day 1 and monitored for a total of 28 days for morbidity, mortality and disease relapse. Mice from each treatment group, untreated and control group were selected to be euthanized, and the bacterial loads in lung and spleen were determined by plating and enumeration of cfu. Untreated mice generally displayed 6 log_10_ cfu in the lung at Day 4 (Figure 1a) and 7 log_10_ cfu in the spleen (Figure 1b). The bacterial burdens detected in the lungs and spleen were consistent with the observed rapid weight loss and 0% survival rate with a median survival of 4 days (Figure 1c and d). The efficacy of TPR1 was assessed at 40 mg/kg and at 80 mg/kg. Both levels of TPR1 dosing demonstrated a reduction in bacterial burden in the lungs and spleen compared with untreated controls. When delivered at 40 mg/kg, TPR1 reduced bacterial growth in the lungs and no bacteria were detected in the spleen (*P *<* *0.01; one-way ANOVA) (Figure 1a and b). The observed partial efficacy of TPR1 delivered at 40 mg/kg is consistent with the observed delayed weight loss and increased median survival of 8 days (Figure 1c and d). Durable cure was achieved when TPR1 was delivered at 80 mg/kg based on no detectable bacteria in the lungs or spleen, reversible weight loss and 100% survival until the study endpoint of 7 × the median survival of untreated controls (Figure 1). This is consistent with the observed differences in efficacy and demonstrates correlations with median survival, bacterial burden in the lungs, control of dissemination and growth in the spleen during treatment.

### TPR1 and doxycycline co-administration at subtherapeutic doses demonstrate efficacy

TPR1 was assessed as a co-therapeutic with the SoC drug doxycycline to determine its use as a new agent in a combination therapy regimen. Dosing at 40 mg/kg doxycycline reduced the bacterial burden by ∼1 log_10_ cfu in the lungs and ∼4 log_10_ cfu in the spleen in comparison to untreated controls (Figure 1a and b), which demonstrates only partial efficacy at this dose. Co-administration of sub-efficacious doses of TPR1 and doxycycline demonstrated efficacy upon treatment withdrawal after 4 days of treatment, and 100% survival until the study endpoint of 28 days, equivalent to ∼7 × the mean time of death of untreated controls (Figure 1c and d). Mice that reached study endpoint were shown to have undetectable bacteria in both the lung and spleen. Importantly, this demonstrates that the combination of TPR1 and doxycycline have an additive effect and are capable of obtaining a ‘durable’ cure even when co-administered at sub-efficacious doses.

In this study, we demonstrated that TPR1 possesses antibacterial activity against a panel of *F. tularensis* strains that includes clinical strains with diverse susceptibilities to current SoC drugs. TPR1 was also shown to have efficacy in the murine model of tularaemia. The improved efficacy of TPR1 alone and in combination with the SoC drug, doxycycline, in the acute model of tularaemia with no observable resistance clearly indicates that this rifampicin derivative has excellent potential as a next-generation chemotherapeutic against tularaemia.
